# Chronic Active Epstein-Barr Virus-Associated Enteritis: CT Findings and Clinical Manifestation

**DOI:** 10.1155/2020/2978410

**Published:** 2020-06-22

**Authors:** Bo Zhang, Xia Wang, Xiaoyan Tian, Yongping Cai, Xingwang Wu

**Affiliations:** ^1^Department of Radiology, The First Affiliated Hospital of Anhui Medical University, Hefei 230022, China; ^2^Department of pathology, The First Affiliated Hospital of Anhui Medical University, Hefei 230022, China

## Abstract

**Aim:**

To improve the identification and computed tomography (CT) diagnostic accuracy of chronic active Epstein-Barr virus (EBV)-associated enteritis (CAEAE) by evaluating its CT findings and clinical manifestation.

**Methods:**

The data of three patients with pathologically and clinically confirmed CAEAE who underwent CT enterography (CTE) were retrospectively reviewed from January 2018 to October 2019. The following data were evaluated: imaging characteristics (length of involvement, pattern of mural thickening, pattern of attenuation, perienteric abnormalities), clinical symptoms, endoscopic records, laboratory examinations, and pathologic findings.

**Results:**

Based on CT findings, two patients demonstrated segmental bowel wall thickening (involvement length >6 cm), asymmetric thickening, layered attenuation, fat stranding, and adenopathy, whereas the remaining one had no positive finding. The endoscopic results of all patients showed numerous irregular ulcers in the colon, and one patient had a focal esophageal ulcer. The major clinical symptoms were abdominal pain (*n* = 3), retrosternal pain (*n* = 1), fever (*n* = 3), diarrhea (*n* = 2), hematochezia (*n* = 1), and adenopathy (*n* = 3). The main laboratory examination indicators were increased serum EBV DNA load (*n* = 1) and increased inflammatory markers (*n* = 3). With regard to the main pathologic findings, all patients showed positive EBV-encoded RNA (EBER) situ hybridization in the colonic biopsy specimen, with one patient being positive in the esophagus.

**Conclusion:**

CAEAE is rare and is usually misdiagnosed as inflammatory bowel disease (IBD). The imaging features of CAEAE overlap with those of Crohn's disease and ulcerative colitis. The presence of segmental and asymmetric bowel wall thickening, layered attenuation, and fat stranding in the CTE image may be helpful in differentiating CAEAE from IBD.

## 1. Introduction

Chronic active Epstein-Barr virus-associated infection (CAEBV) is one of the many subtypes of Epstein-Barr virus (EBV)-associated lymphoproliferative disorders (EBV-LPD) and comprises a range of lymphoid tissue diseases including hyperplastic, borderline, and neoplastic diseases [1]. EBV infection is prevalent and persists as a latent infection [2, 3]. In a few individuals, infected lymphocytes including T, B, and natural killer (NK) cells selectively proliferate into cells with multiple mixed types of clonality, which cause EBV-LPD [4–6]. At present, there are significant regional differences in the distribution of case reports, particularly in East Asian regions, but only a few in the United States and other Western countries [6, 7]. In immunocompetent adults, the incidence of CAEBV infection is quite rare [8], in addition to fever, hepatosplenomegaly, and adenopathy, CAEBV infection often involves multiple organs; the most frequently involved organs are the liver, spleen, bone marrow, lymph nodes, and skin, while the organ system with least involvement is the digestive tract [9].

Chronic active EBV-associated enteritis (CAEAE) was first described in 2005 by Joan Robinson et al. [10]. In recent years, some papers in the literature have reported the clinical and pathological characteristics of CAEAE; however, the detailed imaging characteristics of CAEAE have not been well described [11–14]. Although CAEAE is diagnosed mainly based on the clinical and pathological features of the patient, computed tomography (CT) enterography (CTE) imaging may provide a more evident information on changes in the intestinal morphology that may be different from those of inflammatory bowel disease (IBD). To the best of our knowledge, this study provides the first detailed description of the radiological features of CAEAE.

## 2. Materials and Methods

### 2.1. Study Population

This study was conducted with the approval of the ethics committee of The First Affiliated Hospital of Anhui Medical University, and the requirement for written informed consent was waived in this retrospective study. All cases were obtained from the Department of Pathology and the Department of Radiology at The First Affiliated Hospital of Anhui Medical University in China. From January 2018 to May 2019, the pathology records and medical records of three patients with pathologically and clinically confirmed CAEAE were reviewed. The diagnostic criteria for CAEAE were based on a previous study [15]: (1) recurrent or persistent infectious mononucleosis-like symptom: (a) swelling of lymph nodes, fever, and hepatosplenomegaly; (b) additional complications including digestive tract, hematological, neurological, pulmonary, ocular, dermal, and/or cardiovascular disorders (valvular disease including aneurysm) that mostly have been reported in patients with mononucleosis infection. (2) An unusual pattern of anti-EBV antibodies with elevated anti-EA and anti-VCA and/or detection of increased EBV genomes in affected tissues, including peripheral blood. (3) Chronic disorders that cannot be explained by other known disease processes at diagnosis: hemophagocytic syndrome and T or NK cell lymphoma. The three abovementioned criteria, which no longer emphasize the course of disease >6 months, must be met to establish a diagnosis of CAEBV infection. In addition to meeting these diagnostic criteria, all three patients must have symptoms of the digestive system, with clear endoscopic indications of digestive tract lesions, and histopathologically confirmed positive EBV-encoded RNA (EBER) in situ hybridization in the affected tissues.

### 2.2. CTE Examination

All patients were required to fast overnight prior to CTE examination. The patients achieved adequate bowel distension with the oral administration of 1.5-2 L of iso-osmotic polyethylene glycol solution 1 h before CT scanning.

CTE was conducted using a 64-slice multidetector (Revolution CT, GE Healthcare, Waukesha, WI, USA). The CT scan was performed from the diaphragmatic dome to the symphysis pubis with the following parameters: tube voltage 120 kV, tube current 400 mAs, collimation 0.625 mm × 64 mm, pitch 1.375, and tube rotation speed 0.35 s/c. Prior to the contrast-enhanced scan, a plain CT scan was performed. The following were used for the contrast-enhanced scan: contrast agent Omnipaque (Omnipaque 300; Amersham Health, Princeton, NJ, USA) (300 mg I/mL, 1.5 mL/kg body weight) in the A tube and normal saline in the B tube (50 mL) injected at a rate of 4 mL/sec through the antecubital vein. Arterial phase scanning was performed using an automatic tracking method with a threshold value of 120 HU in the aorta. Intervals of 45 s and 60 s were used for late arterial and venous phase scanning, respectively. The data were reconstructed with a section thickness of 0.625 mm and then transferred to a GE AW 4.7 workstation.

### 2.3. Image Analyses

All CTE images were retrospectively reviewed by two experienced radiologists with 5 and 10 years' experience in CTE, respectively. All image analyses were performed using an AW 4.7 workstation (GE Healthcare), and the radiologists were blinded to the patients' clinical information. Multiplanar reconstruction was performed to visualize and measure the location and morphology of lesions. The following CTE imaging characteristics were evaluated: (1) pattern of mural thickening: a small bowel wall >3 mm or colonic wall >2 mm was defined as bowel wall thickening (asymmetric thickening or symmetric thickening) [16]. (2) Length of involvement: focal involvement (>5 cm), segmental involvement (6-40 cm), or diffuse involvement (>40 cm) [17]. (3) Pattern of attenuation: layered attenuation (a thickened bowel wall with layered alternating densities), white attenuation (the bowel wall is enhanced to a degree equal to or greater than that of venous vessels in the same scan), or grey attenuation (the enhanced bowel wall is similar to that of the muscle on enhanced scans) [18]. (4) Perienteric abnormalities: fat stranding (increased perienteric fat density) and adenopathy.

### 2.4. Clinical Data

Clinical data, including sex, age, clinical symptoms, endoscopic records, laboratory examinations, and pathologic findings, were collected from the electronic medical records.

## 3. Results

### 3.1. Patients' Clinical and Demographic Data

**Figure 1:**
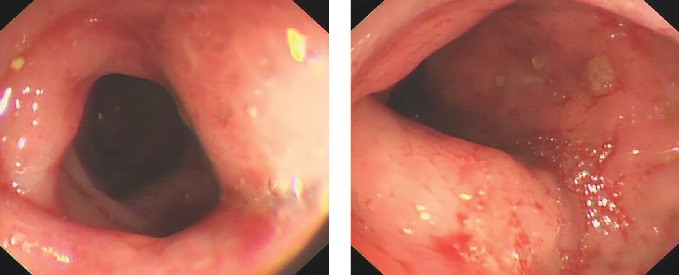
Numerous irregular and shallow ulcers in colon.

**Figure 2:**
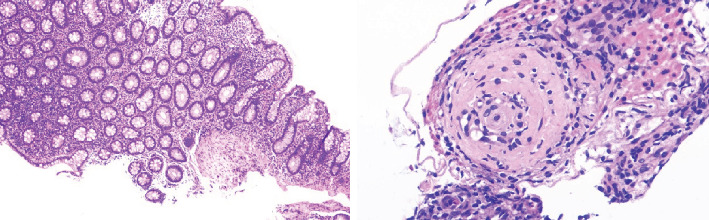
Activity chronic inflammation with ulcer formation in the mucosa, and diffuse inflammatory cell infiltrate within the lamina propria and submucosa, and granulation tissue.

**Figure 3:**
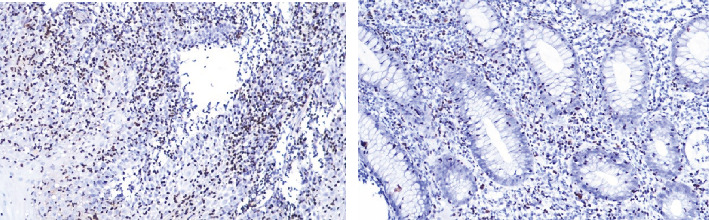
In situ hybridization for Epstein-Barr virus-encoded RNA positive expression in the esophagus and colon.

Among the 3 included patients, there were 2 males and 1 female aged 28-42 years (median age, 39 years). The clinical features and laboratory findings of CAEBV enteritis are summarized in Tables 1 and 2. The major clinical symptoms were abdominal pain (*n* = 3), retrosternal pain (*n* = 1), fever (*n* = 3), diarrhea (*n* = 2), hematochezia (*n* = 1), and adenopathy (*n* = 3). The laboratory findings showed decreased hemoglobin (*n* = 3), increased inflammatory indicators (*n* = 3), evidently increased serum EBV DNA load (*n* = 1), and abnormal coagulation function (*n* = 3). The endoscopic results showed numerous irregular ulcers in the colon (Figure 1). The pathologic findings demonstrated that all patients had chronic inflammation activity with ulcer formation in the mucosa, and a diffuse inflammatory cell infiltrate within the lamina propria and submucosa composed of small-sized lymphocytes with mild atypia (Figure 2). Immunohistochemical stains showed that CD3 was positive in all cases. The results of in situ hybridization for EBER showed that there were more than 50 EBER-positive lymphocytes per high-power field in all cases (three in the colonic biopsy specimen, one positive in the esophagus) (Figure 3).

### 3.2. CTE Findings

**Figure 4:**
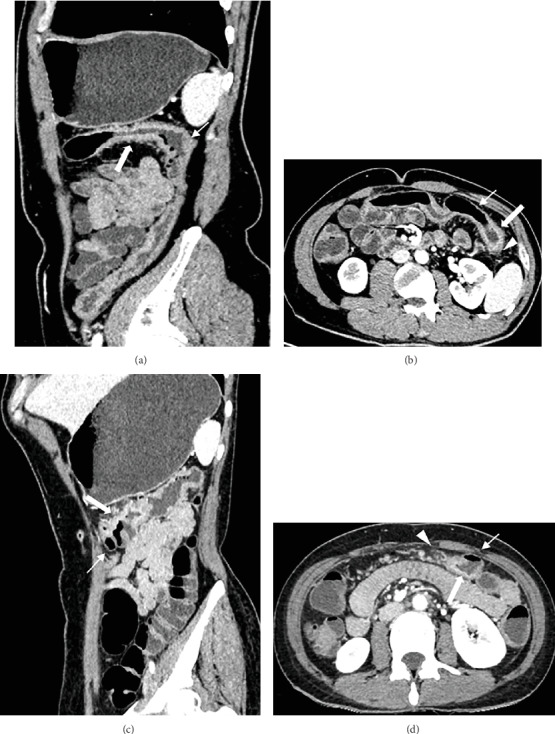
CT enterograph features of case 1 (a, b) and case 3 (c, d): asymmetric thickening (small arrow), layered attenuation (big arrow), fat stranding (triangle).

The CTE features of all cases are summarized in Table 3 and Figure 4. There were no positive findings in one patient. In the remaining patients, thickening of the bowel wall was found mainly in the transverse and descending colons. In terms of the pattern of mural thickening, the maximum thickness of the bowel wall was 11 mm in one patient and 8 mm in another patient, and all of these patients exhibited asymmetric thickening. For the length of involvement, two patients had segmental bowel wall thickening (25 cm, 30 cm). With regard to the pattern of attenuation, two patients had layered attenuation. For perienteric abnormalities, two patients had fat stranding and adenopathy.

## 4. Discussion

EBV is not only prevalent in healthy individuals as a latent infection but also plays an important role in some infectious and neoplastic diseases, such as infectious mononucleosis, Burkitt's lymphoma, NK/T cell lymphoma, and Hodgkin's lymphoma. Besides, EBV has been associated with some lymphoproliferative disorders that may vary between nonneoplastic and neoplastic diseases, such as CAEBV. Primary infections are usually asymptomatic and often occur in infants and young children. Adolescents and adults with EBV infection often manifest self-limited infectious mononucleosis-like symptoms with a good prognosis. However, a few individuals present with chronic or recurrent transmissible mononucleosis, including persistent or intermittent fever with abnormal liver function, hepatosplenomegaly, and adenopathy, accompanied by increased peripheral blood serum EBV DNA load and abnormal changes in EBV antibody [15]. CAEBV usually manifests systemic symptoms; however, gastrointestinal tract symptoms are quite rare. To our knowledge, no more than 50 patients with CAEAE have been reported [11–14], and the studies mainly investigated the clinical and pathological features of CAEAE, but the imaging features were not well described.

In recent years, multislice computed tomography has been developed as an important tool for detecting intestinal lesions, enabling the acquisition of isotropic data and facilitating the ability to perform high-resolution multiplanar reconstruction [19–21]. CTE can effectively show the lesions on the bowel wall and adjacent organs. The small intestine was fully dilated by administering neutral (low-density) oral contrast material, followed by a CT scan of the small intestine phase to maximize the image contrast between the intestinal mucosa and the bowel wall to show the lesions of the intestinal wall more clearly [20]. Based on the findings of this study and the literature review [17–23], the comparison of CTE features between CAEAE and IBD is summarized in Table 4. In this study, we found that the main CTE imaging features of CAEAE patients were colonic involvement, segmental bowel wall thickening, asymmetric thickening, layered attenuation, fat stranding, and adenopathy. One of the patients had no positive CTE finding; a possible explanation for this might be that the gastrointestinal tract is in the early stages of involvement. CAEAE imaging features overlapped with those of Crohn's disease and ulcerative colitis; thus, differentiating CAEAE from IBD could be challenging. Crohn's disease typically involves the terminal ileum and the ascending colon, and ulcerative colitis involves the rectum spreading upward. The imaging characteristics of Crohn's disease consist of discontinuous lesions in the bowel wall (skip areas), prominent vasa recta (comb sign), presence of fistulas and abscesses when the inflammation involves the entire intestinal wall, and proliferation of fats along the mesenteric border of the bowel [20, 23]. The wall thickening in Crohn's disease is usually asymmetric or eccentric because of preferential involvement along the mesenteric border of the intestinal wall; on the contrary, it is symmetric or centric in ulcerative colitis [17]. The typical characteristic of ulcerative colitis is a contiguous superficial lesion in the colon [20, 23].

If the thickness of the bowel is 6-40 cm or >40 cm, it is defined as a segmental or diffuse thickening, respectively [17]. Segmental or diffuse concentric and symmetric thickening of the bowel wall, which is usually no more than 10 mm [19], is typically caused by benign diseases, except for small bowel lymphoma. Concentric and symmetrical thickening of the intestinal wall is generally considered to be a characteristic of benign diseases, such as infection, inflammation, intestinal edema, and ischemia [17]. The exception was that the intestinal associated tumors, such as well-differentiated or small adenocarcinomas, usually manifest symmetric and homogeneous thickening and should be considered particularly when a focal extended thickening in the bowel wall is observed without prominent perienteric fat stranding [22].

Segmental or diffuse thickening is more common in inflammatory and vascular diseases, including ulcerative colitis, infectious microenteritis, hypoproteinemia, and portal hypertension-induced intestinal wall edema and hypoperfusion-induced ischemia [19–21, 23]. A comprehensive evaluation of these two characteristics can narrow the range of differential diagnoses. In this study, we found that CAEAE manifests as a segmental asymmetric or eccentric bowel wall, which could be attributed to malignant tendency [1]. Layered attenuation normally suggests benign lesions, such as acute Crohn's disease, vasculitis, intestinal wall edema, and ischemic bowel wall due to a variety of causes, usually to strengthen the inner layer and outer layer, respectively, reflecting inflammation or ischemia in the mucosal and serosal layer; intermediate and low density represents the submucosal layer of the edema [20, 21, 23]. As the CAEAE progressed, the accompanying transmural inflammation increases the density of perienteric fat. We found that CAEAE causes slight fat stranding similar to Crohn's disease and ulcerative colitis. By contrast, in some inflammatory conditions such as epiploic appendagitis, diverticulitis, appendicitis, and omental infarction, the inflammatory changes in the perienteric mesentery and fat are more serious than in the bowel wall [24]. Nevertheless, CAEAE has the characteristics of both Crohn's disease, including asymmetric and fat stranding, and ulcerative colitis, such as segmental thickening and involvement of the transverse and descending colons. Hence, the diagnosis of CAEAE still needs to be confirmed with clinical data.

In terms of clinical data, we found that the clinical symptoms were abdominal pain, fever, hepatosplenomegaly, diarrhea, hematochezia, and adenopathy, consistent with other reports [11–14]. Retrosternal pain was first reported in our study. A possible explanation for this clinical symptom may be esophageal involvement. CAEAE has been considered to be associated with viral replication. Serum EBV DNA load can be used as a monitoring indicator of the severity of CAEAE and treatment effect. At present, the most sensitive detection method is artificial in situ hybridization of EBER with a sensitivity of almost 100%, which is considered to be the gold standard for confirming EBV infection [25]. However, the number of EBER-positive cells detected within the tissue showed no correlation with the number of EBV DNA copies in the peripheral blood or the histologic findings [26]. Similarly, we found that one of the patients was EBER positive, whereas the serum EBV DNA load was very low. In a few conditions, EBER-positive lymphocytes were detected in more than 60% of patients with IBD, particularly in those with ulcerative colitis [27, 28]. Most of these patients have nonsystemic EBV infection without symptoms such as fever and hepatosplenomegaly and lymph node enlargement; such EBV infection is considered to be a type of opportunistic infection, wherein the role of IBD is unclear.

With regard to endoscopic findings, we found there were no integrative continuous mucosal damage, cobble-like appearance, and longitudinal ulcers; however, there were numerous irregular, variously sized ulcers in the colon, mucosal erythema, edema, and erosion accompanied by purulent secretion, while in patients with ulcerative colitis, endoscopy typically revealed a uniform, continuous inflammation extending proximally from the rectum, but limited to the colon [29]. These findings are consistent with those of previous reports [11–14]. On the contrary, typical endoscopic changes of Crohn's disease included segmentally distributed longitudinal ulceration and skip lesions in the mucosa, while pathological biopsy showed noncaseous granuloma lesions [29]. Pathologic findings consisted of chronic inflammation activity with ulcer formation in the mucosa and the presence of granulomatous tissue and diffuse inflammatory cell infiltrate within the lamina propria and submucosa composed of small-sized lymphocytes with mild atypia.

CAEAE and IBD are similar in endoscopic and pathological morphology. The key to differential diagnosis is that CAEBV infection usually presents with fever or hepatosplenomegaly, and adenopathy, with rare manifestations of ulcerative colitis such as purulent stool and tenesmus. Compared with ulcerative colitis, diffuse disturbed crypt architecture and crypt abscesses are rare. Although CAEAE may also present with a transmural inflammatory fissure ulcer and occasionally granulomatous structure, which is easily confused with Crohn's disease, it generally lacks typical chronic interstitial changes such as granuloma, mucosal muscle hyperplasia, and nerve hyperplasia and hypertrophy [14].

The treatment of CAEBV infection is difficult. Antiherpesvirus drugs such as acyclovir and ganciclovir do not respond to active EBV infection. Glucocorticoid immunosuppressants (cyclosporine), immunomodulators, and cytotoxic chemotherapy drugs (cyclophosphamide, anthracyclines, vincristine, etoposide, and prednisone) can provide short-term relief from active EBV infection, but there is no cure. Hematopoietic stem cell bone marrow transplantation is a surgical treatment method for CAEBV infection; however, there are also risks associated with transplant complications [30].

## 5. Conclusions

In summary, the differential diagnosis of CAEAE from IBD may be difficult, because these two conditions share similarities in terms of imaging, clinical signs, endoscopic manifestations, laboratory examinations, and pathology. The presence of segmental and asymmetric bowel wall thickening, layered attenuation, and fat stranding in the CTE image may provide some valuable information to help differentiate CAEAE from IBD.

## Figures and Tables

**Table 1:** Clinical data of the three CAEAE patients.

Case	Sex	Age	Clinical symptoms	EBV DNA (copies/mL)	Organ involvement	HB (g/L)	Inflammatory indicators	Coagulation function	Misdiagnose
1	M	39	Abdomin al pain, fever, adenopathy, diarrhea, hematochezia	<5 × 10	Colon	12	Increased	Altered	UC
2	M	28	Abdominal pain, fever, adenopathy, retrosternal pain, diarrhea, splenomegaly	3.8 × 104	Colon, esophagus	92	Increased	Altered	IBD
3	F	42	Abdominal pain, fever, adenopathy,	NA	Colon	105	Increased	Altered	CD

M: male; F: female; UC: ulcerative colitis; IBD: inflammatory bowel disease; CD: Crohn's disease.; NA: not available.

**Table 2:** Endoscopic and pathologic findings of the three CAEAE patients.

Case	Endoscopy	Biopsy samples	HE stain	Immunohistochemistry	EBER
1	Numerous irregular ulcers in the colon	Hepatic flexure of colon, transverse colon, descending colon, sigmoid colon	Lymphatic follicles in the lamina propria, crypt abscess in some glands, aggregation of atypical lymphoid cells	CD3, CD7+ Ki-67+<5%	Colon positive
2	Numerous shallow and small ulcers in the colon	Ascending colon, transverse colon, descending colon, rectum, esophagus, throat	Tissue granulation, atypical lymphocyte infiltration	Colon:CD2, CD3, CD4, CD8, TIA-1, GrB+. Ki-67(+, 20%). Throat:CD2, CD3, CD7, CD56(+). Ki-67(+, 40%).	Colon and esophagus positive
3	Numerous irregular ulcers in the colon	Ileocecal junction, ascending colon, transverse colon, descending colon	Granulomatous tissue and lymphoid tissue hyperplasia, atypical lymphocyte infiltration	CD3, CD20, Pax-5, CD4, CD8(+), Ki-67(+, 20%).	Colon positive

**Table 3:** CTE findings of the three CAEAE patients.

Case	Pattern of mural thickening	Length of involvement	Pattern of attenuation	Perienteric abnormalities
1	11 mm^a^,	25 cm	Layered attenuation	Fat stranding,
	Asymmetric thickening			Adenopathy
2	NF	NF	NF	NF
3	8 mm^a^,	30c m	Layered attenuation	Fat stranding
	Asymmetric thickening			Adenopathy

^a^Maximum thickness; NF: not found.

**Table 4:** Comparison of CTE features between CAEAE and IBD.

	Main involved location	Pattern of mural thickening	Length of involvement	Pattern of attenuation	Perienteric abnormalities
CAEAE^a^	All segmental colons may be involved	Asymmetric thickening	Segmental involvement	Layered attenuation	Fat stranding, adenopathy
CD^b^	Terminal ileum, ascending colon	Asymmetric thickening	Focal involvement	Layered or white attenuation (acute, active disease), grey attenuation (chronic disease)	Fat stranding, adenopathy, prominent vasa recta, fistulas and abscesses
UC^b^	Rectum, descending colon	Symmetric thickening	Segmental or diffuse involvement	Layered attenuation	Not common

^a^Based on our study findings.

^b^Common and typical CTE features based on literature review.

## Data Availability

The data used to support the findings of this study including the CTenterography images, and the clinical data are available from the corresponding author upon request.
